# Labial Adhesion—An Uncommon Adult Condition: Clinical Features, Management and Histopathological Findings—A Case Series Study

**DOI:** 10.3390/life16050757

**Published:** 2026-05-01

**Authors:** Iulia Mihaela Gavrila, Elena Cristina Burlacu, Doina Iulia Nacea, Raluca Tatar, Andrei Marin, Andreea Ungureanu, Silviu-Adrian Marinescu, Carmen Giuglea

**Affiliations:** 1 Department of Plastic Reconstructive Surgery, Faculty of Medicine, University of Medicine and Pharmacy “Carol Davila”, 020021 Bucharest, Romania; 2 Department of Plastic Reconstructive Surgery, “Saint John” Clinical Emergency Hospital, 042122 Bucharest, Romania; 3 Department of Plastic Reconstructive Surgery and Burns, “Grigore Alexandrescu” Clinical Emergency Hospital for Children, 011743 Bucharest, Romania; 4 Department of Plastic Reconstructive Surgery, “Bagdasar Arseni” Clinical Emergency Hospital, 041915 Bucharest, Romania; andreea.ungureanu2020@drd.umfcd.ro

**Keywords:** labial coalescence, labial adhesion, adult, treatment, histopathology, classification

## Abstract

Background: Labial adhesion in adult women is a rare condition characterized by the complete or partial fusion of the labia minora. According to a 2024 literature review, only 112 cases have been documented over a 38-year period. Methods: We performed a retrospective study, analyzing the files of all patients admitted to the Plastic Surgery Department of “Saint John” Hospital in Bucharest, Romania, over the last 12 years for any cases of labial adhesion. From the medical files, we summarized demographic data, clinical presentation, risk factors, comorbidities, surgical findings, outcomes, follow-up and histopathology results. During the investigated period, we identified three patients admitted to our hospital with labial adhesion with ages between 67 and 79 years. All of them had complete labial fusion with symptomatic complications at the time of diagnosis, and all three required surgery. Tissue samples were collected from all selected patients and sent for histopathological examination, which highlighted several different types of changes. All three cases had a follow-up period of one year. Conclusions: Labial coalescence in adult women is a rare condition more commonly associated with the postmenopausal period. All patients presented with complete labial coalescence, making surgical intervention necessary as topical therapy was not an option. Histopathological examination was crucial to confirm the diagnosis. These results provide important clinical insights and contribute to the limited literature on this rare condition.

## 1. Introduction

Labial adhesion, or labial fusion, is a rare condition in adult women defined by the partial or complete fusion of the labia minora. Such a disorder can obstruct the vaginal introitus and, in more severe cases, even the external urethral opening. The cases of labial coalescence in women reported in the literature are rare compared to prepubertal girls [1,2,3].

Factors related to this condition are hormonal changes (especially hypoestrogenism), acute or chronic inflammatory processes, local trauma (such as sexual assault, accidental falls, or blunt trauma), dermatological conditions (herpes simplex, pemphigoid, lichen sclerosus, or systemic lupus erythematosus), caustic vaginitis and inadequate hygiene [4]. Surgery is necessary in cases where topical therapy, most often estrogen or corticosteroids, fails to treat the problem [5].

In adult women, labial coalescence can significantly compromise quality of life. The patients usually present to the doctor when the adhesion is complete or symptomatic. Symptoms include local distress or pain, especially during walking or prolonged sitting. Sexually active patients experience dyspareunia and an inability to have sexual intercourse [6]. Urinary complications are common and may include recurrent infections and urinary retention [7].

However, this disorder remains largely underdiagnosed in adult women, with most published data focusing on pediatric manifestations [8]. A recent systematic review of international databases spanning 38 years (1985–2023) identified only 112 cases in the adult population after excluding duplicates and pediatric reports [1]. Most of the papers on this topic are of case report type or short case series and are centred on clinical and local treatment. In addition to these, in this paper, all the presented cases are complemented by a detailed description of the lesions from the histopathological point of view. The histopathological description is an important element in formulating a complete diagnosis and establishing the mechanisms of the occurrence of labial fusion.

The aim of this study is to describe the clinical presentation, management, and outcomes of labial coalescence in adult women treated in our institution. At the same time, the novelty that our article brings is a classification of labial coalescence in adult women, a classification that does not currently exist.

## 2. Materials and Methods

We performed a retrospective study, analyzing the files of all patients admitted to the Plastic Surgery Department of “Saint John” Hospital in Bucharest, Romania, over the last 12 years for any cases of labial adhesion. Cases involving patients under 18 years were excluded.

From the medical files, we summarized demographic data, clinical presentation, risk factors, comorbidities, surgical findings, outcomes, follow-up and histopathological results.

This study was approved by the Ethics Committee of the St. John Emergency Clinical Hospital, No. R14509/29.08.2025. Informed consent was obtained from all patients included in the study, and patient confidentiality was maintained. All data were anonymized in accordance with ethical principles and patient confidentiality regulations. Each case is presented individually in a logical structure, based on the data collected for this study.

## 3. Results

During the investigated period, we identified three women admitted to our hospital for labial adhesion with ages between 67 and 79 years. All three patients were well-groomed, maintained good local hygiene, and were sexually inactive. All cases had complete labial fusion at the time of diagnosis.

**Case 1**

**Figure 1:**
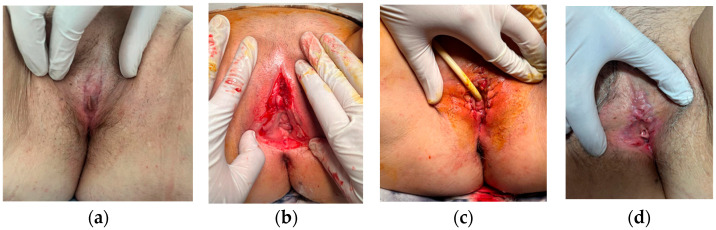
(**a**) Clinical appearance before surgery—total coalescence of labia minora. (**b**) Intraoperative findings: After surgical incisions were made along the labia minora, (**c**) immediate postoperative appearance, (**d**) appearance at 4 weeks postoperation.

A 67-year-old postmenopausal patient attended a medical consultation for marked vulvar discomfort, burning sensations, and urinary retention. The patient’s medical history indicated hypothyroidism in treatment with levothyroxine (Euthyrox) and a synovial cyst of the right hand, surgically treated five years ago, without any previous vulvar pathology reported. The urinary symptoms progressively worsened over two years, culminating in complete obstruction. Clinical examination revealed complete labial fusion, with total coverage of the clitoris, external urethral meatus and vaginal introitus (Figure 1a). Preoperative assessment was within normal limits. Preoperative care consisted of administering 0.6 mL of low-molecular-weight heparin (LMWH).

Surgery was performed under spinal anesthesia. Bilateral skin incisions were made along the labia minora, encompassing the fused tissue, which was excised and sent for histopathological analysis. Once the urethral opening was exposed, a Foley catheter (CH 18) was inserted. Upon releasing the clitoris, a dense, whitish secretion was noted and sampled for culture and sensitivity. The result of bacteriological examination was negative. After antiseptic lavage and careful hemostasis, the labia minora were anatomically reconstructed with absorbable interrupted sutures (Figure 1b,c). A separate lesion on the vaginal mucosa was excised and submitted for histopathologic examination. The patient remained hospitalized for three days. Antibiotics (first-generation cephalosporin) were administered intraoperatively and on the first day postoperation. The urinary catheter was maintained for one day after the surgery. Anti-inflammatory drugs were administered for 5 days. Medical checkup was performed weekly in the first month and after that every 3 months during the first year (Figure 1d).

**Figure 2:**
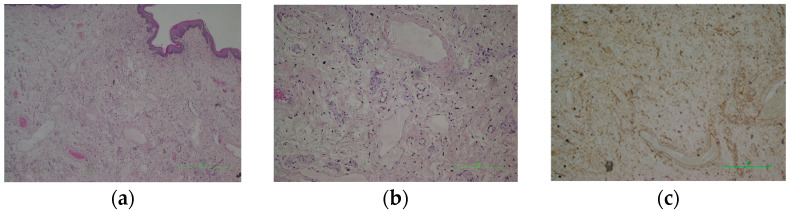
(**a**) Hematoxylin–eosin stain; 4× objective—Hypocellular mesenchymal cell proliferation. (**b**) Hematoxylin–eosin stain; 10× objective—Spindle and stellate cells with minimal atypia, embedded in a myxoid matrix, with medium- and large-calibre vessels. (**c**) CD34—Positive.

Histopathology findings showed a hypocellular mesenchymal tumour with an infiltrative pattern composed of spindle and stellate cells with minimal nuclear atypia within a myxoid stroma (Figure 2a,b). Vessels of varying calibres were present, along with hemorrhagic extravasation and minimal lymphoplasmacytic infiltration. Immunohistochemistry: Desmin—focally positive; CD34—focally positive (Figure 2c); SMA—focally positive; S100—negative in tumour cells, positive in nerve fibres. Final diagnosis: Aggressive angiomyxoma. The excised vaginal mucosal lesion was diagnosed as a fibroepithelial polyp.

**Case 2**

**Figure 3:**
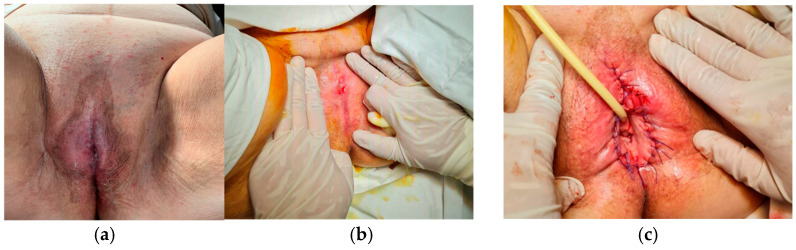
(**a**,**b**) Clinical appearance before surgery—total coalescence of labia minora. (**c**) Immediate postoperative appearance.

A 79-year-old postmenopausal woman with no significant comorbidities presented with complete urinary retention. During a prior urological consultation, catheterization was attempted, at which point complete labial fusion was noticed (Figure 3a). Surgical separation of the labia minora was performed under spinal anesthesia, followed by a Foley catheter (CH 18) insertion and specimen collection for histopathological analysis (Figure 3b,c). Intraoperatively, a thick, yellow, foul-smelling vaginal discharge was observed and sampled for microbiological analysis. Cultures identified *Klebsiella* spp., multisensitive. Antibiotics (second-generation cephalosporin) were administered intraoperatively and on the first day after surgery. The patient was treated with oral Augmentin for 10 more days after discharge. The Foley catheter was maintained for one day postoperation. She was hospitalized for four days and returned weekly during the first month and every three months until the end of the second year.

**Figure 4:**
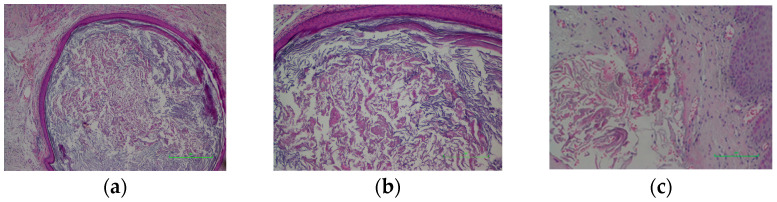
(**a**). Hematoxylin–eosin stain; 4× objective—Cyst lined by squamous epithelium. (**b**) Hematoxylin–eosin stain; 10× objective—Keratin filament content. (**c**) Hematoxylin–eosin stain; 20× objective—Multinucleated giant cells associated with keratin filaments.

Histopathological examination revealed adipose and neurovascular fibroconjunctival tissue partially covered by stratified squamous epithelium with areas of compact hyperkeratosis and mild-to-moderate acanthosis. Focal cystic spaces delimited by keratinizing squamous epithelium (Figure 4a) with granular layers filled with lamellar keratin (Figure 4b) were observed. Areas of cystic wall destruction were associated with granulation tissue and rare multinucleated giant cells (Figure 4c), along with extensive areas of fibrous restructuring. Final diagnosis: Fractured epidermoid cyst with chronic fibrosing inflammatory process.

**Case 3**

**Figure 5:**
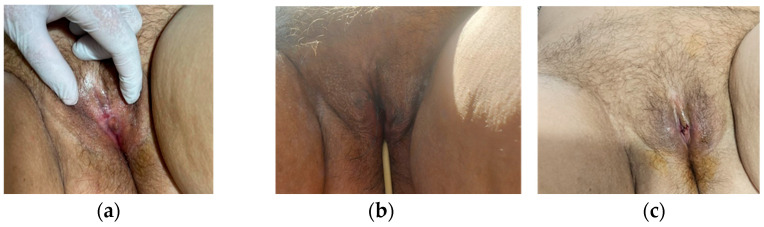
(**a**) Clinical appearance before surgery—total coalescence of labia minora. (**b**) Immediate postoperative appearance. (**c**) Appearance at 4 weeks postoperation.

A 72-year-old postmenopausal woman with essential hypertension and chronic peripheral venous insufficiency attended a medical consultation for complete fusion of the labia minora (Figure 5a). At admission, she reported burning sensations and significant vaginal discomfort. Surgical treatment was performed under spinal anesthesia. She received antibiotics (second-generation cephalosporin) intraoperatively and on the first day postoperation.

A midline incision was made above the clitoris, exposing the clitoris and external urethral meatus. Then, a Foley catheter (CH 18) was inserted and maintained for one day postoperation. A V-shaped excision of the fused labial tissue was performed, followed by hemostasis, antiseptic irrigation, and skin closure using 3-0 absorbable sutures (Figure 5). The excised tissue was sent for histopathology analysis. The patient required 6 days of hospitalization.

**Figure 6:**
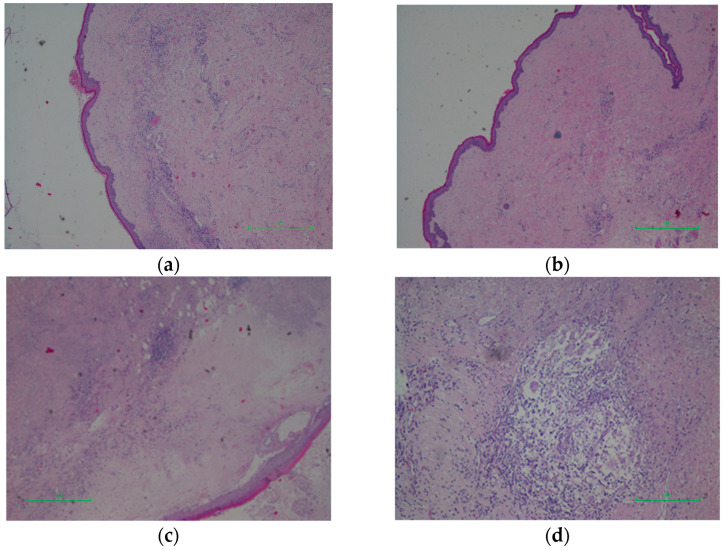
(**a**) Hematoxylin–eosin stain; 4× objective—Atrophic epithelium; sclerosis in the lamina propria. (**b**) Hematoxylin–eosin stain; 4× objective—Atrophic epithelium; sclerosis in the lamina propria. (**c**) Hematoxylin–eosin stain; 4× objective—Edema in the papillary dermis; hyalinization; moderate inflammatory infiltrate. (**d**) Hematoxylin-eosin stain; 10× objective—Chronic inflammatory process with lymphocytes, plasma cells, histiocytes, and multinucleated giant cells.

Histopathological examination indicated atrophic epithelium (Figure 6a,b) with hyperkeratosis, sclerosis in the lamina propria, edema in the papillary dermis, hyalinization of the lamina propria and a moderate chronic inflammatory infiltrate (Figure 6c) composed of lymphocytes, plasma cells, histiocytes, and multinucleated giant cells (Figure 6d). Final diagnosis: Lichen sclerosus.

## 4. Discussion

The Plastic Surgery Department of the “Saint John” Hospital diagnosed and treated only three women for labial adhesion across the 2012–2024 period. They were admitted between August 2023 and July 2024. A potential explanation for the previously low number of presentations may be that patients with partial labial coalescence tolerated the symptoms and did not seek medical attention until more severe complications occurred.

Complications arising from labial coalescence may be physical or psychological. The main symptoms for which the patients in our case series sought treatment were complete urinary retention and local soreness. Other clinical manifestations reported in the literature are urinary infections [9], hematuria, dysuria [10,11], pyosalpinx [12], and urinary incontinence [13,14,15]. Psychological effects are poorly documented, although several case studies suggest significant emotional consequences: feelings of inferiority, anxiety, sadness, frustration, guilt, shame, social isolation, and reduced quality of life [16]. Proper genital examination is crucial for accurate diagnosis, and a lack of such assessment may lead to prolonged psychological distress [17].

Regarding the labial coalescence classification, to the best of our knowledge and based on a comprehensive literature review, only one categorization has been published, and it applies exclusively to pediatric cases. It was proposed in 2020 and includes the following four subtypes [18]:

Type I (48%)—Thin, translucent adhesions that can be easily separated manually.

Type II (20%)—Thick adhesions obscuring the vestibular structures.

Type III (24%)—Thick adhesions with hymenal involvement adherent to the labia minora.

Type IV (8%)—Lateralized fusion (right or left), not on the midline.

This classification was cited in only one subsequent publication in 2021 [19].

There is no specific classification for adult women in the current literature, so we propose the following classification of labial coalescence in adult women, based on our clinical observations—the Gavrila Classification:

G I—Incomplete anterior labial fusion in which the labia minora fuse anteriorly, covering the clitoris and possibly the external urethral meatus.

G II—Incomplete posterior labial fusion in which the labia minora fuse posteriorly, covering the vulvar vestibule.

G III—Complete labial fusion with a small residual opening through which urine can pass.

G IV—Complete labial fusion without a residual opening, resulting in complete urinary obstruction.

In G I and II, adult women (especially older women) may not seek medical attention due to the tolerability of symptoms. However, in G III and IV, complications such as urinary retention or recurrent infections would compel patients to seek medical evaluation. For the last two types, surgery would be the main therapeutic option.

Labial adhesion is usually associated with pediatric patients. The incidence in the pediatric population is estimated to be 1.8% with a peak in the age range of 13–23 months [20]. More recently, the literature has begun to recognize its occurrence in adults, mainly in postmenopausal women, as a result of estrogen deficiency and chronic inflammation [21]. Estrogen deficiency is the common point between prepubertal girls and postmenopausal women. The real prevalence of labial adhesion in the adult population is currently unknown, as this pathology is underdiagnosed in adult women. The most recent and comprehensive review of this pathology was published by Mahmoudnejad et al. [1]. This review investigated all available studies on the subject and found that almost all were isolated case reports or small case series, most involving only one to three patients. The authors emphasized that there are no standardized diagnostic criteria or evidence-based treatment protocols, that the therapeutic approach often varies greatly between physicians and that there is a lack of long-term outcomes. So, it is difficult to estimate the risk of recurrence or the effectiveness of treatment over time. These findings confirm that labial coalescence in postmenopausal women remains an under-researched and underdiagnosed condition. Future research should focus on multicenter, prospective studies that could establish prevalence, risk factors, and effective interventions. Underreporting may be due to the sensitive nature of vulvar symptomatology, both intimately and socially. Embarrassment, stigma, or minimization of genital symptoms often delay presentation to a physician [22,23]. In addition, many routine gynecological visits do not include a thorough vulvar examination, leading to underestimation.

Talking about treatment, the first choice for labial coalescence in children is topical estrogen application [8]. Although topical estrogen therapy may be effective in cases related to vulvovaginal atrophy due to estrogen deficiency, the lack of clinical improvement after an adequate treatment period should raise suspicion for alternative underlying conditions, including lichen sclerosus or autoimmune vulvar disorders. In symptomatic situations in adult women, estrogen therapy can be effective within 6–12 weeks, but surgery is the standard of care for the management of labial adhesion [4,15,24]. Due to the fact that all patients had complete labial fusion with difficulty urinating, we could not wait 6–12 weeks for estrogen treatment. Rapid intervention was necessary. In all three cases, surgery was performed under spinal anesthesia. Even though spinal anesthesia seems to be the best anesthesia option for the surgical treatment of this pathology, there have been publications in which surgical separation of the labia was performed under local [4] or general anesthesia [25].

Surgical intervention consists of the careful separation of fibrous adhesions. It is essential to protect the surrounding structures, especially the urethra and clitoris. Meticulous dissection is necessary to minimize mucosal trauma and avoid postoperative scarring, especially in postmenopausal patients with fragile and atrophic mucosa [17]. If any discharge was observed during adhesiolysis, samples were collected for bacteriological examination. A Foley urinary catheter was introduced during the surgery and removed after 24 h. All the patients received antibiotics intraoperatively.

Samples from the fused tissue were collected from all the analyzed cases and sent for histopathological examination. Histopathological examination plays a crucial role in differentiating benign labial fusion from various underlying neoplasms. Also, in cases of labial adhesions in postmenopausal women, histopathological analysis is essential to identify chronic inflammatory dermatoses, such as lichen sclerosus, which require long-term treatment and surveillance [26]. In some cases, the exact cause of labial fusion may remain unknown, even when histological analysis is performed, highlighting the complexity of this condition [24]. Despite its potential diagnostic value, histologic analysis is scarcely performed or systematically described in existing studies on adult women with labial fusion.

Aggressive angiomyxoma is a rare mesenchymal tumour that frequently affects the vulvovaginal region [27]. Since its initial description in 1983, only approximately 250 cases had been reported globally by 2009 [28]. Typically, it presents as a large mass [29], rather than the flat lesion identified in our case. Moreover, it generally does not cause labial coalescence. It is known for its high local recurrence rate, ranging between 36% and 72% following surgical excision [30]. In our case, there has been no evidence of recurrence after two years of follow-up.

Epidermoid cysts, which are benign subepidermal nodules filled with keratin [31], do not usually cause labial fusion per se. However, in our second case, chronic inflammation may have contributed to the development of labial adhesions.

The most frequent cause of labial coalescence in adult women remains dermatologic disease, particularly lichen sclerosus [1,4,4,32], as seen in our third patient.

We believe that there is a critical gap regarding the histopathological correlation with labial fusion: without certain diagnoses, physicians may overlook inflammatory or autoimmune etiologies or even preneoplastic processes.

A standardized clinical classification has not been proposed, leaving adult labial fusion without a histology-informed diagnostic framework.

This gap in the literature regarding labial coalescences points to several critical directions for future research. First, observational studies involving postmenopausal women in gynecological, dermatological, urological, and plastic surgery clinics could provide valuable data on prevalence. Second, some diagnostic criteria that can be standardized should be established to help differentiate labial coalescence from other vulvar pathologies. Third, clinical trials comparing outcomes after hormonal treatments and after surgery could help identify best practices for symptom resolution and recurrence prevention.

In our case series, two of the three patients had conditions that suggest an immune-mediated background, like lichen sclerosus and synovial cyst. It is known that lichen sclerosus is a chronic inflammatory dermatosis with autoimmune mechanisms, defined by lymphocytic infiltration, epithelial atrophy, and progressive sclerosis of the vulvar tissue. This may stimulate fibrotic reorganization and adhesions between the labia. In some cases, immune-mediated mechanisms may contribute to labial adhesions. In select cases, a multidisciplinary evaluation, including rheumatology, may be helpful [33]. In the differential diagnosis of labial adhesions in adult women, autoimmune conditions like Sjögren syndrome and localized scleroderma should be considered. These autoimmune diseases may follow similar pathways of chronic inflammation and tissue fibrosis. These processes may contribute to architectural changes in the vulva and may promote labial adhesions. Therefore, a multidisciplinary evaluation, including rheumatological analysis, may be necessary to exclude an underlying autoimmune condition [34,35].

In addition to surgical treatment, topical immunomodulatory therapies may be considered in inflammatory vulvar conditions associated with labial adhesions. Calcineurin inhibitors such as tacrolimus or pimecrolimus can be used, particularly in lichen sclerosus resistant to corticosteroids, by reducing local inflammation. More recently, JAK inhibitors like tofacitinib have shown potential in immune-mediated skin diseases; however, their use in vulvar disorders is still limited and requires further study [36,37].

A careful differential diagnosis is essential in adult patients presenting with labial fusion. The most common causes are vulvovaginal atrophy due to estrogen deficiency, especially in postmenopausal women, and chronic inflammatory conditions such as lichen sclerosus. Less commonly, autoimmune disorders like mucous membrane pemphigoid can affect the vulvar mucosa and contribute to adhesion formation through chronic inflammation and scarring.

Clinically, vulvovaginal atrophy usually presents as thin, pale, and fragile mucosa, often accompanied by dryness, irritation, and dyspareunia, and typically responds well to topical estrogen therapy. In contrast, lichen sclerosus often appears as porcelain-white plaques with epithelial atrophy, sclerosis, and progressive distortion of vulvar architecture. Because these conditions can have similar features, histopathological examination is essential in cases with atypical presentation or insufficient response to conservative treatment, ensuring an accurate diagnosis and appropriate management [38]. If no significant improvement is observed after up to twelve weeks of treatment, further evaluation—including a vulvar biopsy—should be considered to rule out other conditions such as lichen sclerosus or autoimmune blistering diseases.

This study has several limitations that should be noted. First, the retrospective study design may introduce some errors and limit the ability to create causal relationships. Second, the limited number of patients—only three cases—restricts the extrapolation of the results. Third, this study was conducted at a single centre, which may not adequately represent the diversity of clinical presentations. Potential immune-mediated mechanisms were considered based on clinical findings; however, not all patients underwent immunological or rheumatological evaluations, which limits definitive conclusions about pathophysiology. Even with these limitations, our study offers important information on the clinical and histopathological aspects of this rare condition and may help improve the management of labial coalescence in adult patients.

Given the global ageing of the population and the increase in longevity among women, this condition may become more frequent in clinical practice.

There is an urgent need for greater awareness among clinicians and research efforts directed towards increasing the quality of life of postmenopausal women affected by labial coalescence.

## 5. Conclusions

All patients presented with complete labial coalescence, which necessitated surgical treatment. The clinical presentation in these cases did not allow for topical therapy, making surgical intervention essential. Histopathological examination was essential to confirm the diagnosis, revealing a variety of changes in the tissue collected from the area of labial coalescence. This case series indicates that labial coalescence in elderly women is a rare condition associated with sexual inactivity. Intraoperative placement of a urinary catheter was performed to ensure safe surgical management and prevent urethral injury. Postoperative management included antibiotic therapy to reduce the risk of infection and careful clinical monitoring to assess healing and functional outcomes. These findings highlight the importance of a multidisciplinary approach, combining surgical, pharmacological, and clinical strategies in the management of this rare condition. Given the limited number of reported cases in the literature, our findings provide additional clinical insight into the management of this rare condition.

Given the major differences between children and adults who develop labial adhesion, we consider the introduction of a classification framework of this pathology dedicated exclusively to adults to be necessary and useful for current medical practice.

## Data Availability

All relevant data and findings are presented within this article. For additional information or specific inquiries, interested readers are encouraged to contact the corresponding author.

## Abbreviations

The following abbreviations are used in this manuscript:

LMWH	Low-Molecular-Weight Heparin
CH 18	Charriere 18
spp.	Species
